# Heartbreakers or Healers? Innate Lymphoid Cells in Cardiovascular Disease and Obesity

**DOI:** 10.3389/fimmu.2022.903678

**Published:** 2022-05-11

**Authors:** Luke B. Roberts, Graham M. Lord, Jane K. Howard

**Affiliations:** ^1^School of Immunology and Microbial Sciences, King’s College London, London, United Kingdom; ^2^Faculty of Biology, Medicine and Health, University of Manchester, Manchester, United Kingdom; ^3^School of Cardiovascular and Metabolic Medicine & Sciences, King’s College London, London, United Kingdom

**Keywords:** ILCs, innate lymphoid cells, cardiovascular disease, obesity, heart

## Abstract

Cardiovascular diseases (CVDs) are responsible for most pre-mature deaths worldwide, contributing significantly to the global burden of disease and its associated costs to individuals and healthcare systems. Obesity and associated metabolic inflammation underlie development of several major health conditions which act as direct risk factors for development of CVDs. Immune system responses contribute greatly to CVD development and progression, as well as disease resolution. Innate lymphoid cells (ILCs) are a family of helper-like and cytotoxic lymphocytes, typically enriched at barrier sites such as the skin, lung, and gastrointestinal tract. However, recent studies indicate that most solid organs and tissues are home to resident populations of ILCs - including those of the cardiovascular system. Despite their relative rarity, ILCs contribute to many important biological effects during health, whilst promoting inflammatory responses during tissue damage and disease. This mini review will discuss the evidence for pathological and protective roles of ILCs in CVD, and its associated risk factor, obesity.

## Introduction

Cardiovascular diseases (CVDs) are the major global cause of premature human death. Obesity and associated metabolic inflammation underlie the development of several major health issues including type 2 diabetes (T2DM), insulin resistance, hypertension, dyslipidaemia, and increased expression of inflammatory mediators - all of which are risk factors for CVDs. Tackling obesity therefore remains a key goal the effort to reduce the increasing burden of CVDs worldwide.

Five major subsets of Innate lymphoid cells (ILCs) are acknowledged: cytotoxic natural killer cells (NKs), lymphoid tissue inducers (LTi) and helper-like ILC1s, ILC2s and ILC3s (1). ILC1s depend on the transcription factor (TF) T-bet for their development (2) and peripheral maintenance (3). They produce IFNγ, tumor necrosis factor (TNF) (4, 5), and TGF-β under some contexts (6). ILC2s are regulated by a suite of TFs including GATA3 and RORα, express type 2 cytokines including interleukin (IL)-4, IL-5, IL-9 and IL-13, and tissue repair factors including amphiregulin. LTi and helper-like ILC3s express TF RORγt and produce IL-17, IL-22, and TNF-superfamily members, including lymphotoxins. ILCs are activated by environmental signals including cytokines, tissue-derived danger signals, metabolites, neurotransmitters, and neuropeptides (1, 7).

While NKs are predominantly circulatory, helper-like ILCs reside in tissues and are enriched at barrier mucosal sites such as the lung and intestinal tract. However, since their discovery, almost all organs have been found to play host to ILC subsets, including tissues of the cardiovascular system (8). Cardiovascular-associated ILCs (cILCs) reside in the pericardium and pericardial fluid (9), in the adventitia of arteries including the aorta (10), and in fat-associated lymphoid clusters (FALC) (11) of perivascular (12) and pericardial adipose tissue (13). The majority of cILCs are ILC2s or ILC2-commited precursors, with minimal presence of ILC1s and ILC3s (14, 15). As observed in other tissues, cILC2 populations are dependent on IL-33 signalling for their development as well as their activation and function. cILC2s may even be more responsive to IL-33 signalling than counterparts at barrier sites such as the lungs, due to unique phenotypic attributes including greater expression of GATA3 (15). Cardiac fibroblasts are a main source of IL-33 in the heart, responsible for ILC2 homeostasis and activity (9), while adventitial stromal cells provide IL-33 and thymic stromal lymphopoietin (TSLP) to ILC2s in arteries and FALC, providing a tissue supportive niche for their development and activation (16). Developmental, adoptive transfer, and parabiosis studies in mice suggest that, similarly to ILCs from barrier sites (17–19), cILC populations are tissue-resident cells, seeded during early embryonic development and shortly after birth, sustaining themselves through local renewal, even during cardiac inflammation (14, 15).

**Figure 1:**
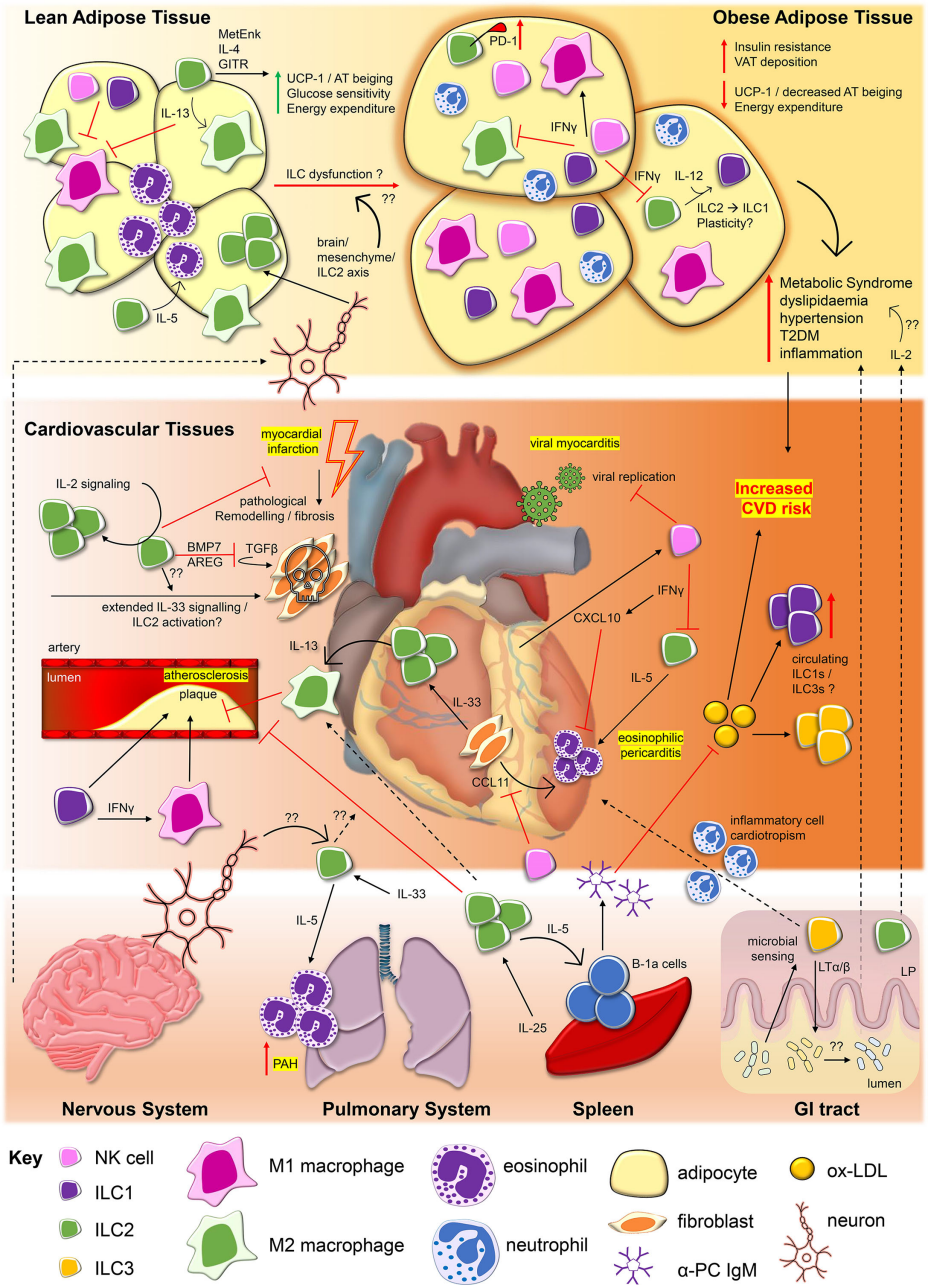
Innate lymphoid cells contribute to protective and pathological processes in cardiovascular diseases and obesity. During viral myocarditis caused by agents such as Coxsackie virus B3 (*CV-B3*), NKs restrict viral replication (20) and are recruited to cardiac tissues by cardiomyocyte production of C-X-C motif chemokine ligand 10 (*CXCL10*), itself promoted by NK-derived interferon gamma (*IFNγ*) (21). IFNγ may also restrict ILC2 activity, influencing ILC2 capacity to drive inflammation associated with eosinophilic pericarditis. Interleukin (*IL*)-33 derived from cardiac fibroblasts drives cardiac ILC2 proliferation and along with fibroblast production of eotaxins (i.e. eotaxin-1/*CCL11*), ILC2 production of IL-5 may facilitate recruitment of eosinophils to the pericardium (9). ILC2 production of IL-13 can also promote polarisation of M2 macrophages, which may serve protective roles in *atherosclerosis* (12, 22). Conversely, ILC1s and promotion of classically activated proinflammatory M1 macrophages may promote atherosclerotic plaque formation (23–25). ILC2s, regulated by IL-2 signalling, may also serve protective roles for cardiac tissue repair following major adverse events such as myocardial infarction, *via* the production of amphiregulin (AREG) and bone morphogenic protein 7 (*BMP7*), inhibiting pathological tissue remodelling and fibrosis (26). The impact of prolonged activation of ILC2s by factors such as IL-33 on the outcome of ILC2 repair responses in this context require further study (27). In the circulation, increased frequencies of ILC1s and ILC3s are associated with major cardiovascular and cerebrovascular events including ST-elevated myocardial infarction (STEMI) and acute cerebrovascular infarction (ACI/Stroke) (28, 29). This may be related to increased circulating levels of oxidised low-density lipoproteins (*ox-LDL*) in patients, but the impact of these alterations to ILC subset frequencies inpatients remain to be elucidated. *Obesity*, and its associated metabolic inflammation is associated with major risk factors for the development of CVDs, including type 2 diabetes mellitus (*T2DM*), dyslipidaemia, hypertension, and inflammation. As observed in cardiovascular tissues, ILC2s are the dominant ILC subtype found within *lean adipose tissue*. ILC2s promote alternatively activated M2 macrophage phenotypes and eosinophils within adipose tissue *via* their production of IL-5 and IL-13, contributing to lean adipose tissue homeostasis (30–33). ILC2 expression of methionine enkephalin (*MetEnk*) (31) and IL-4 (34), in addition to signaling *via* Glucocorticoid-induced TNFR-related protein (*GITR*) (32) can protect from obesity by promoting beiging of white adipose tissue, linked to upregulation of mitochondrial uncoupling protein 1 (UCP-1), increasing glucose sensitivity and energy expenditure. In *obese adipose tissue* ILC2 numbers and functions are impaired, contributing to increased visceral adipose tissue (*VAT*) depots, insulin resistance and decreased beiging. Upregulation of inhibitory receptor PD-1 by ILC2s (35), negative regulation by IFNγ derived from ILC1s and natural killer cells (NKs) (36, 37), and ILC2 to ILC1 conversion (38) may be among the mechanisms which result in ILC2 dysfunction in obesity. NKs and ILC1s numbers are also altered in obesity, potentially impacting upon the ratio between inflammatory M1 and anti-inflammatory M2 macrophages (33, 39). Influences from physiological signals and ILCs present in other tissues may also impact upon CVDs and obesity pathogenesis. The *nervous system* can regulate ILC2 activity within lean adipose tissue *via* a brain/mesenchymal/ILC2 axis (40). Dysregulation of this axis may contribute to development of obesity, however, broader effects of neuronal signalling on ILCs in the context of CVDs requires further study. In the induction of pulmonary arterial hypertension (*PAH*), IL-5 derived from *pulmonary system* ILC2s are responsible for tissue eosinophilia which may drive arterial damage (41). IL-25 drives ILC2 proliferation in the *spleen*, promoting atheroprotective effects, IL-5-dependent B-1a expansion and production of anti- phosphorylcholine (PC) Immunoglobulin M (*IgM*) which targets ox-LDL (42, 43). In the *gastrointestinal (GI) tract*, sensing of microbial composition by ILC3s may influence inflammatory cell cardiotropism in the context of myocarditis (44), while ILC2s and ILC3s within the intestinal lamina propria (LP) may also contribute to processes driving obesity, through factors such as production of IL-2 (45).

This mini review will summarise the known protective and pathological actions of ILCs in the context of CVDs. As a major cardiometabolic risk factor, obesity, and its relatedness to ILC activity will also be discussed. **Figure 1** summarises the information presented. **Table 1** provides a list of the abbreviations used throughout.

**Table 1:** Meanings of abbreviations used in this article.

Abbreviation	Meaning
ACI	Acute cerebrovascular infarction
AS	Atherosclerosis
AT	Adipose tissue
AT1-ILCs	Group 1 adipose tissue innate lymphoid cells
ATM	Adipose tissue macrophage
BAT	Brown adipose tissue
cILCs	Cardiovascular-associated innate lymphoid cells
CVD/CVDs	Cardiovascular disease/s
DCM	Dilated cardiomyopathy
DIO	Diet-induced obesity
EAM	Experimental autoimmune myocarditis
FALC	Fat-associated lymphoid tissue
HFD	High fat diet
ILC/ILCs	Innate lymphoid cell/innate lymphoid cells
ILC1/ILC1s	Helper-like type 1 innate lymphoid cell/s
ILC2/ILC2s	Helper-like type 2 innate lymphoid cell/s
ILC3/ILC3s	Helper-like type 3 innate lymphoid cell/s
LTi	Lymphoid tissue inducer cell
MI	Myocardial infarction
NK/NKs	Natural killer cell/natural killer cells
ox-LDL	Oxidised low-density lipoprotein
PAH	Pulmonary arterial hypertension
PBMCs	Peripheral blood mononuclear cells
PC	phosphorylcholine
STEMI	ST-elevation myocardial infarction
T2DM	Type 2 Diabetes mellitus
TF/TFs	Transcription factor/s
TSLP	Thymic stromal lymphopoietin
WAT	White adipose tissue

## Protective and Reparative Roles of ILCs in CVD

### NK Cells

Viral myocarditis is predominantly caused by infection with Coxsackievirus B3. NKs limit viral replication primarily through their potent cytotoxicity, among other protective mechanisms (20). NKs are recruited to the infected heart by cardiomyocyte upregulation of CXCL10, a chemokine ligand of the NK-expressed receptor CXCR3 (21). Expression of CXCL10 is promoted by NK-derived IFNγ - a feed-forward mechanism, driven by NKs for further expansion of the population with in the tissue (21).

Observational studies linking decreased NK numbers and activities, with low grade inflammation and increased disease severity, implicate a protective role for NK cells in atherosclerosis (AS) and coronary artery disease (CAD) (46–48). These effects may be related to NK apoptosis (49) and increased expression of inhibitory molecules (50). However, results from murine models complicate interpretation of these findings, suggesting either pro-atherogenic roles for NKs (51), or conversely, no impact on AS development (52).

NKs may also serve cardioprotective functions during eosinophilic myocarditis, restricting eosinophil influx and survival. Anti-asialo GM1-mediated NK depletion in an anti-MHC (myosin heavy chain) immunisation model of experimental autoimmune myocarditis (EAM) resulted in worsened disease outcome and enhanced eosinophilic influx, accompanied by greater cardiac tissue fibrosis (53). NKs may defend against eosinophilic inflammation directly, by promoting eosinophil apoptosis (36, 53, 54), and indirectly by suppressing fibroblast production of the eosinophil recruiting chemokine eotaxin-1 (CCL11). Furthermore, CXCL10 is an inhibitor of eosinophil recruitment *via* antagonism of the eosinophil trafficking receptor CCR3 (53, 55). IFNγ also restricts ILC2 cytokine expression and limits the active niche in which ILC2s can exert their effects (56, 57). As will be discussed, ILC2s may promote pathological recruitment of eosinophils in the setting of cardiac inflammation (9). IFNγ production by NKs may therefore also act to inhibit cardiac ILC2 activity.

### ILC2s

Apolipoprotein E deficient mice (*Apoe*^-/-^) display defective lipoprotein clearance and accrue abnormal levels of low-density lipids, making them prone to development of AS. IL-25 administration limits initiation and progression of AS in high fat diet (HFD)-fed *Apoe*^-/-^ mice. This is concomitant with expansion of splenic ILC2 populations and enhanced IL-5 production (42, 43). Furthermore, IL-25 treatment increases levels of circulating anti-phosphorylcholine (PC) IgM, dependent on intact IL-5 expression, indicative of an atheroprotective effect of ILC2 activation by IL-25. The PC epitope is a component of oxidised low-density lipoprotein (ox-LDL), strongly associated with AS development. Anti-PC IgM is produced by B-1a cells – an atheroprotective, innate-like B cell subtype. B-1a cells expand following IL-25 treatment (43), and depend on IL-5 for their survival and maturation to produce natural IgM antibodies (58, 59). Transfer of IL-25-expanded, wild type ILC2s to *Apo*e^-/-^ mice reduces the lipid content of AS lesions and augments B-1a cells and anti-PC IgM, suggestive of a therapeutic avenue to tackle AS (42). Of note, the ILC2/IL-5/B-1a axis is critically dependent on ILC2 expression of the helix loop-helix TF, ID3 (60). As an ID3 single nucleotide polymorphism (rs11574) is associated with increased carotid intimal media thickness, this may link ILC2 dysfunction in humans with enhanced AS risk (61).

HFD feeding also reduces peripheral ILC2 numbers and alters their functional cytokine responses in AS-prone mouse strains - linking defective ILC2 activity with disease (12). Low-density lipoprotein receptor deficient mice (*Ldlr^-/-^*) share a similar functional defect in lipid clearance as *Apoe*^-/-^ mice and are similarly AS-prone. *Ldlr^-/-^* mice reconstituted with bone marrow from ILC2 deficient mice (^Staggerer^/*Rora*^Flox^ x *Il7ra*^Cre^) displayed accelerated AS plaque lesion development. This was associated with reduced collagen deposition found in larger plaques (indicative of increased plaque destabilisation) and altered plaque immune cell composition, dependent on loss of ILC2-derived IL-13. Plaque macrophage phenotypes were disrupted by ILC2 loss, with reduced arginase-1 expression suggestive of a restricted capacity for tissue repair. This supports work identifying IL-13 as an atheroprotective cytokine, acting *via* induction of alternatively activated (M2) macrophages (22), potentially supporting the augmentation of ILC2 responses as a novel therapeutic strategy for AS. Indeed, expansion of CD25^+^ ILC2s by IL-2/anti-IL-2 complexes reduces circulating ox-LDL and AS lesion development in HFD-fed lymphopenic *Rag*^-/-^*Ldlr*^-/-^ mice (10), indicating that IL-2 therapy may be a novel treatment option in some forms of CVD. Supportive of this, genetic ablation of ILC2s in a murine model of myocardial infarction (MI) delayed recovery of cardiac function. MI-protective functions of ILC2s were associated with an upstream regulatory IL-2 signalling axis (13). Furthermore, analysis of peripheral blood from patients recruited to a placebo-controlled, double-blind trial designed to assess the safety of Low-dose InterLeukin-2 treatment in patients with stable ischaemic heart disease and Acute Coronary Syndromes [LILACS trial (62)], revealed a short-term increase in circulating ILC2s, serum IL-5, and eosinophil counts (13). This indicates that IL-2 therapy activates human ILC2s *in vivo*. However, putative protective roles of ILC2s in this context remain to be fully investigated. Post-cardiac injury, augmentation of ILC2 repair responses may also be an attractive therapeutic option, inhibiting TGF-β1-driven pathological fibrosis and remodelling by cardiac fibroblasts which can lead to terminal heart failure (26).

### ILC3s

ILC3 associated cytokines IL-17A and IL-22 have been linked to CVDs, but may be protective under certain contexts (63–65). ILC3s might also be protective during hepatic (66) and intestinal (67) ischemia-reperfusion-injury (IRI), suggestive, potentially, of similar roles during reperfusion following MI. However, in both the liver and the intestinal tract, ILC3s are far more populous than in cardiovascular tissues, where Th17 cells are also more likely to be the major source of ILC3-associated effector cytokines. Whether ILC3s serve protective roles in CVDs requires further investigation.

## ILCs and CVD Pathogenesis

### ILC1s

ILC1s constitute only a minor population of the ILCs identified within murine cardiovascular tissues (14, 15). Despite this, *Ldlr*^-/-^ and *Apoe*^-/-^ models combining genetic knockout of T-bet or IL-12, implicate roles for ILC1s in development of HFD-induced AS (23, 24). However, these models also deplete other cells important in AS pathogenesis, including Th1 and NKs (68). ILC1s have been more specifically linked to AS plaque development using adaptive lymphocyte deficient *Rag*^-/-^*Apoe*^-/-^ models. Anti-NK1.1 or anti-IL-15R antibodies were used to deplete ILC1s and/or NK cells respectively, followed by adoptive transfer of splenic ILC1s, identifying a possible role for ILC1s in aggravation of AS (25).

Involvement of ILC1s in the pathology of major cardiovascular and cerebrovascular adverse events has been proposed, based on correlations between disease severity and circulating frequency of these cells. Total number and proportions of ILCs among circulating leukocytes were increased in acute ST-segment elevation myocardial infarction (STEMI) patients, compared to healthy controls (28). This was accounted for by a specific increase in ILC1s, while numbers of ILC2s and ILC3s remained unaltered, indicating an expansion of ILC1s rather than ILC subset plasticity towards the ILC1 phenotype (69, 70). Similar observations were made in acute cerebral infarction (ACI) patients (29). A significantly increased proportion of ILC1s among total circulating ILCs, profiled at the time of admission, was observed compared to healthy controls. Concomitant with this was a decrease in the proportion of ILC2s and no impact on circulating ILC3s. However, as total number of ILCs among leukocytes was not reported, whether alterations to subset prevalence among total ILCs represents phenotypic plasticity between ILC2s to ILC1s (38, 71–73) in the context of ACI, or reflects a specific expansion of ILC1s, remains unclear.

### ILC2s

ILC2s may drive the recruitment of eosinophils to the pericardium *via* production of IL-5 and promotion of cardiac fibroblast-derived eotaxins (9). Choi et al., utilised repeated IL-33 administration to induce eosinophilic pericarditis in mice, finding that pericardial IL-33R^+^ (ST2^+^) ILC2s greatly expanded in response to treatment (9). IL-5 deficiency prevented pericardial eosinophil infiltration, however *Il13*^-/-^ mice displayed a similar level of pericarditis to WT mice. Furthermore, total ILCs were enriched in the pericardial fluid of pericarditis patients, relative to patients with other heart diseases or healthy controls. However, deeper analysis of ILC subsets was not reported, impeding specific association of human pericarditis with ILC2s. ILC2-derived IL-5 also promotes eosinophil accumulation around pulmonary arteries, following chronic activation by IL-33 (41). This accumulation resulted in severe arterial occlusion and pulmonary arterial hypertrophy (PAH). This was ablated in IL-5 deficient and eosinophil deficient mice, but not in *Rag*^-/-^ mice, supporting an ILC2/IL-5/eosinophil dependent axis in the aetiology of PAH. Furthermore, PAH has previously been associated with vascular remodelling driven by IL-33 (74). Despite eosinophilia and other abnormalities in IL-5 overexpressing mice, pulmonary arteries in these animals do not appear to be affected (75). Therefore, while the ILC2/eosinophil axis plays a role, other factors also contribute to PAH development. Prolonged IL-33/ST2 signalling may also contribute to poorer cardiac remodelling and promote heart failure following MI (27), despite augmenting ILC2-driven type 2 immune activity, which may be protective or reparative of ventricular function in the acute phase post-MI (57). ILC2s therefore act as a ‘‘double-edged sword’’ (37), the activity of which must be precisely balanced to provide protection, while limiting pathological outcomes.

### ILC3s

Few studies have investigated ILC3s in CVD pathogenesis. During EAM, differential disease susceptibility in mice from different sources has been associated with intestinal ILC3s and specific microbiome profiles (44). Anti-CD90 antibody mediated depletion of ILCs restricted inflammatory leukocyte trafficking to the heart during EAM, indicating that microbial sensing *via* ILC3s plays a role in development of inflammatory heart diseases *via* cardiotropic immune cell chemotaxis. Additionally, a study investigating ILC3s in axial spondyloarthritis (axSpA) indirectly suggests ILC3 differentiation is promoted by risk factors driving CVD (76). Patients with inflammatory joint conditions, including axSpA, have an increased risk of developing CVD (77). Therefore, clinical management of CVD risk factors, including dyslipidaemia, is important. Circulating IL-22^+^ ILC3s were increased in axSPa patients with dyslipidaemia compared to patients without dyslipidaemia or healthy controls. Furthermore, ox-LDL promoted IL-22^+^ and IL-17^+^ ILC3 differentiation from axSPa patient peripheral blood mononuclear cells (PBMCs) (76) and ILC3s expressed the ox-LDL receptor CD36, blockade of which prevented ox-LDL-mediated differentiation of the cells (76). This indicates that ox-LDL affects ILC3 generation, similar to observations made for ILC1 differentiation from ACI patient PBMCs (29). Although in that study, no effect of ox-LDL stimulation on ILC3s was observed, suggestive of other disease-specific effects of ox-LDL signalling on ILCs. ILC3s are regulated by hypoxia, acting *via* hypoxia inducible factor (HIF)-1α (78). Tissue hypoxia can be induced by, and contribute to CVDs (79). Hypoxia-driven ILC3 responses might therefore also play a role in some forms of CVD.

### ILCs, Adipose Tissue and Obesity

Lean adipose tissue (AT) contains populations of immune cells important for maintaining metabolic homeostasis, including regulatory T cells (T_REGs_), eosinophils and M2 macrophages. These cells are regulated by ILC2s resident in AT-depots in mice and humans (30–33) and these functions are reliant on IL-33 signalling (30, 33). Disruption of IL-33 signalling in mice reduces ILC2s in white AT (WAT), increasing visceral fat depots, impairing glucose homeostasis, and disrupting energy metabolism by prevention of WAT beiging (33). Beiging converts white adipocytes, or adipogenic precursors within WAT, to brown-like AT and is associated with the upregulation of mitochondrial uncoupling protein 1 (UCP1). This increases thermogenesis - raising body heat and calorific expenditure, protecting from obesity, at least in mice. Mechanistically, IL-33-mediated beiging is dependent on the ILC2-expressed opioid peptide methionine enkephalin, which acts on WAT to induce UCP1 expression (31), similarly to the actions of other ILC2-associated molecules like IL-4 (34) and activation of Glucocorticoid-induced TNFR-related protein (GITR), expressed by both human and murine AT ILC2s (32).

During obesity in humans and in murine diet-induced obesity (DIO) models, AT ILC2 frequencies are reduced, and their functions become dysregulated. This may negatively impact the homeostatic, immunometabolic roles of ILC2s, such as restricting differentiation of obesity-protective M2 AT macrophages (ATM) (30). The mechanisms responsible for reduction of ILC2 responses during obesity are likely multifaceted and remain under study. Recent developments suggest they include the upregulation and activation of inhibitory co-stimulatory receptor PD-1 on ILC2s during obesity (35), and dysregulation of brain-AT circuits, which modulate ILC2 activation indirectly *via* neuro-mesenchymal interactions (40). Furthermore, as human and murine AT is also populated by group 1 ILCs (AT1-ILCs), including mature and immature NKs, and non-NK ILC1s, AT ILC2 activities may be affected by aberrant IFNγ signaling, which restrains ILC2 responses (30, 56). An increase in AT ILC1s during DIO has been reported by some studies. This might drive ATM M1 polarization *via* IFNγ production, promoting insulin resistance and metabolic dysfunction in a manner dependent on IL-12 and downstream STAT4 signalling (80). IL-12-mediated plasticity of AT ILC2s to ILC1-like cells may further contribute to these effects (38). ATM M1 responses may also be promoted by increased WAT expression of CD36 in the context of defective ILC2 functions, resulting in enhanced absorption of saturated fatty acids by adipocytes, driving M1 polarisation (33). Conversely, Boulenouar and colleagues report that obesity is associated with a decrease in the number and cytotoxicity of AT1-ILCs. This may contribute to ATM accumulation and alterations to the ratio of proinflammatory M1 to anti-inflammatory M2, thereby impacting insulin resistance and metabolic dysfunction (39). The contribution of AT1-ILC responses and their roles in AT homeostasis and during obesity is an area that requires further investigation.

While AT ILC2s have emerged as important metabolic regulators which promote AT homeostasis, ILC2s from other tissues may also act to promote obesity. In the setting of lymphopenic DIO murine models, adoptive transfer of small intestinal ILC2s, but not WAT ILC2s, promotes obesity *via* an axis dependent on their production of IL-2 (45) – supportive of a role for this cytokine in obesity induction (81). Intestinal ILC3s also moderately contributed to DIO (45). Currently, few studies have investigated the roles of ILC3s in obesity. However, increased frequency of circulating ILC3s in children who are overweight and asthmatic (compared to children who are asthmatic but not-overweight) suggests that obesity may be an independent risk-factor for promotion of ILC3 differentiation (82). Furthermore, the lymphotoxin pathway may promote DIO by driving a pro-obesity intestinal microbiota (83). As LTi and helper-ILC3s produce lymphotoxins, this potentially implicates these cells in obesity pathogenesis.

## Conclusions and Future Perspectives

CVD and obesity are major global health concerns. ILCs are emerging as cells capable of both driving or protecting from CVD pathology. As the field expands, it will shed light on current gaps in our knowledge – for instance, whether ILC1s or ILC3s serve any CVD protective functions. Studies which investigate whether and how cILCs are regulated by factors known to control ILC activity in other tissues and disease states are also required. This may include investigation of cILC regulation by neurotransmitters and neuropeptides (1, 84), post-transcriptional regulation by non-coding RNAs (85–87), and effects of metabolic dysfunction, such as lactic acidosis (88) - a known indicator of cardiac stress. Further studies which compare functions and transcriptional profiles of cILCs with more widely studied barrier ILC populations would also be useful. This would facilitate understanding of similarities and unique differences between these populations, related to the pressures and requirements exerted by the disparate tissue environments these cells function within. Such work may inform new, tissue-specific therapeutic strategies. Encouragingly, there are now several studies which demonstrate how modification of ILC responses might show promise for therapeutic intervention of CVD and obesity in humans. However, most of our mechanistic knowledge about ILC activity is still derived from murine models. As these models are often lymphopenic in nature, the potential translational meaning of these observations to immunocompetent human disease settings is somewhat confounded. Future research should continue to explore and unravel cardiovascular-associated ILC responses, particularly focusing on their roles in the context of human disease.

## Author Contributions

LR conceptualised the manuscript’s theme. LR researched and wrote the manuscript. LR designed and created the figures. GL and JH advised on content and edited the manuscript. All authors contributed to the article and approved the submitted version.

## Funding

LR is supported by the National Institute for Health Research (NIHR) Biomedical Research Centre based at Guy’s and St Thomas’ NHS Foundation Trust and King’s College London. JH is supported by the Medical Research Council (grant MR/K002996/1). GL is supported by the Wellcome Trust (grant number 091009), the British Heart Foundation (award number PG/12/36/29444), the MRC (grant number MR/M003493/1), and the NIHR at Guy’s & St Thomas’ NHS Foundation Trust in partnership with King’s College London and King’s College Hospital NHS Foundation Trust.

## Conflict of Interest

The authors declare that the research was conducted in the absence of any commercial or financial relationships that could be construed as a potential conflict of interest.

## Publisher’s Note

All claims expressed in this article are solely those of the authors and do not necessarily represent those of their affiliated organizations, or those of the publisher, the editors and the reviewers. Any product that may be evaluated in this article, or claim that may be made by its manufacturer, is not guaranteed or endorsed by the publisher.
